# Artificial Intelligence: A Next-Level Approach in Confronting the COVID-19 Pandemic

**DOI:** 10.3390/healthcare11060854

**Published:** 2023-03-14

**Authors:** V. Mahalakshmi, Awatef Balobaid, B. Kanisha, R. Sasirekha, M. Ramkumar Raja

**Affiliations:** 1 Department of Computer Science, College of Computer Science & Information Technology, Jazan University, Jazan 45142, Saudi Arabia; 2 Department of Computer Science and Engineering, School of Computing, College of Engineering and Technology, SRM Institute of Science and Technology, Chengalpattu 603203, India; 3 Department of Computing Technologies, SRM Institute of Science and Technology, Kattankulathur Campus, Chengalpattu 603203, India; 4 Department of Electrical Engineering, College of Engineering, King Khalid University, Abha 62529, Saudi Arabia

**Keywords:** artificial intelligence, COVID-19, nanofiber mask, face mask, coronavirus, AI law

## Abstract

The severe acute respiratory syndrome coronavirus 2 (SARS-CoV-2) which caused coronavirus diseases (COVID-19) in late 2019 in China created a devastating economical loss and loss of human lives. To date, 11 variants have been identified with minimum to maximum severity of infection and surges in cases. Bacterial co-infection/secondary infection is identified during viral respiratory infection, which is a vital reason for morbidity and mortality. The occurrence of secondary infections is an additional burden to the healthcare system; therefore, the quick diagnosis of both COVID-19 and secondary infections will reduce work pressure on healthcare workers. Therefore, well-established support from Artificial Intelligence (AI) could reduce the stress in healthcare and even help in creating novel products to defend against the coronavirus. AI is one of the rapidly growing fields with numerous applications for the healthcare sector. The present review aims to access the recent literature on the role of AI and how its subfamily machine learning (ML) and deep learning (DL) are used to curb the pandemic’s effects. We discuss the role of AI in COVID-19 infections, the detection of secondary infections, technology-assisted protection from COVID-19, global laws and regulations on AI, and the impact of the pandemic on public life.

## 1. Introduction

**Figure 1:**
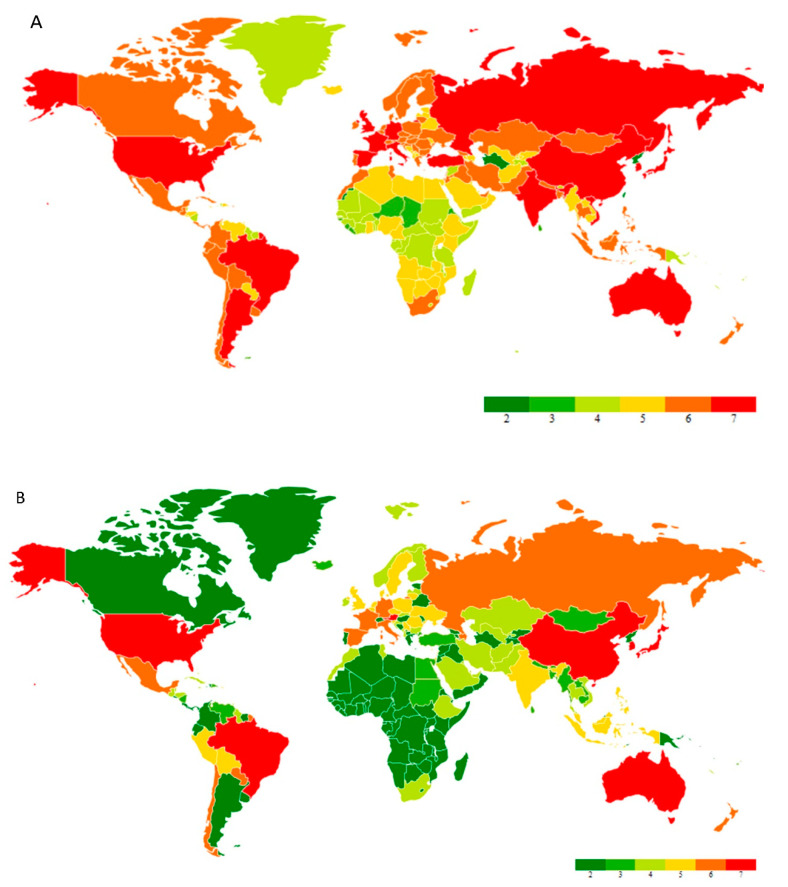
(**A**) Global heat map of total confirmed cases of coronavirus as of 17 January 2023. (**B**) A global heat map of confirmed cases of coronavirus from the past 7 days (11 January 2023 to 17 January 2023). Note: The map was created in Microsoft Excel using the Geographic Heat Map add-in application (Keyur Patel).

The pandemic emerged from Wuhan city, Hubei province in China. It was colloquially known as coronavirus and later named SARS-CoV-2, which caused the disease COVID-19, reported by the World Health Organization (WHO) at the end of 2019 [1]. Since the first case was reported in Thailand, away from mainland China on 13 January 2020, in a short time COVID-19 cases reached various countries and made governments helpless against an invisible enemy. The WHO estimated that 4291 people’s lives were lost with an alarming level of spreading and severity of positive cases of up to 118,000 in 114 counties until 11 March 2020, when with a deep assessment, the WHO declared COVID-19 as a pandemic [2]. As of 14 November 2022, 631 million positive cases have been identified with 6.58 million deaths recorded to date, along with 12.8 billion doses of vaccines administered globally [3]. COVID-19 management has become a key factor in various countries, such as the United States of America, China, the United Kingdom, Italy, France, Germany, Russia, India, Brazil, etc. COVID-19 infected enormous numbers of people in the United States of America, France, Germany, India, Turkey, Italy, Spain, and the United Kingdom. A global heat map image exhibits the cumulative cases globally to date and cases recorded in the past seven days (Figure 1).

Hence, it is worth thinking about what brought on the pandemic. Humans have experienced much from the previous outbreaks that occurred in the past century, such as the bubonic plague in the 14th century, which killed nearly 50 million and lasted up to 5 years [4]; the Spanish flu outbreak in World War I, which killed 50–100 million [5]; and so on. However, the recent pandemics of SARS, MERS, and COVID-19 in 2003, 2012, and 2019 were caused by viruses belonging to the coronavirus family, and have more similarities in their modes of infection. The uniqueness of COVID-19 is its rapid spreading, which is human-to-human transmission, and nearly 20% of infected people are symptomless and spread the infection as carriers known as “super-spreaders” [6,7]. According to recent findings, immunocompromised people, elderly people, patients with cancer, diabetes mellitus, heart diseases, and HIV/AIDS patients are much more susceptible to COVID-19 [8].

The outbreak and global transmission of SARS-CoV-2 was due to the lack of accurate information at an early stage as well as knowledge of future transmission prediction. Therefore, before the governments realized and initiated the containment measures, COVID-19 spread to various countries where people did not have self-protection awareness measures such as wearing masks, social distancing, etc. Countries with a limited healthcare infrastructure suffered more due to a lack of vaccines, systematic treatment, and elevated hospital bills that were not affordable [9]. Still, there is no effective antiviral drug; patients are treated with general treatment methods such as bed rest, ventilators, and conventional antibiotics. Moreover, few antiviral drugs such as remdesivir, nirmatrelvir, bebtelovimab, molnupiravir, and convalescent plasma therapy are approved by the Food and Drug Administration (FDA) in the USA for emergency use for COVID-19 [10].

Secondary infections occurring during COVID-19 treatment are not new to healthcare, as secondary infections occurred during previous pandemics. Serological evidence from SARS patients indicated the presence of *Chlamydophila pneumoniae* and *Mycoplasma pneumoniae* co-infections [11]. MERS-CoV-infected patients were reported to have influenza, tuberculosis, and bacteria and viral co-infections [12]. Similarly, COVID-19 has brought about a variety of secondary infections and co-infections in patients. Many reports exhibited that secondary infections such as mucormycosis [13,14,15], blood coagulation [16,17], blood stream infections [18,19], urinary track infections (UTI) [20,21,22], and antibiotic resistance [23,24,25,26] were reported in COVID-19 patients during hospitalization or occurred post-infection. The coronavirus infection initially affects the lungs and disrupts the epithelial cell, leading to organ failure. Viral and drug-triggered immunosuppression elevated the secondary infections, which brought morbidity and mortality in serious cases [27]. To combat the current situation, advanced computational methods are mandatory to confront the effects of the pandemic.

Recently, Artificial Intelligence (AI) has received more attention due to its ability to observe, make decisions about, and interpret a huge volume of data in different fields [9], which include computer science, healthcare, drug discovery, engineering, infectious diseases, and government management, especially in this pandemic. Modern medicine and improvements in the healthcare sector have brought a huge amount of patient data related to medications. Over the last three decades, researchers have developed and proposed various support systems for the diagnosis and medical treatment of diseases to reduce the burden on healthcare workers [28]. The recent advancement and implementation of mathematical tools in disease diagnostics, treatment, drug targeting, and epidemic prediction with available data aid in disease outbreak control. Various analytical tools have been proposed in recent times, and Artificial Intelligence (AI)-based models are highly valued and promising [29]. In the recent past, AI-based tools have been successfully implemented in various arbovirus-related epidemic diseases such as Dengue, Chikungunya, and Zika, which affect a large population in the South American continent [30], as well as the Middle East respiratory syndrome (MERS) [31], and Ebola [32] which affect the Middle East and African continent.

**Figure 2:**
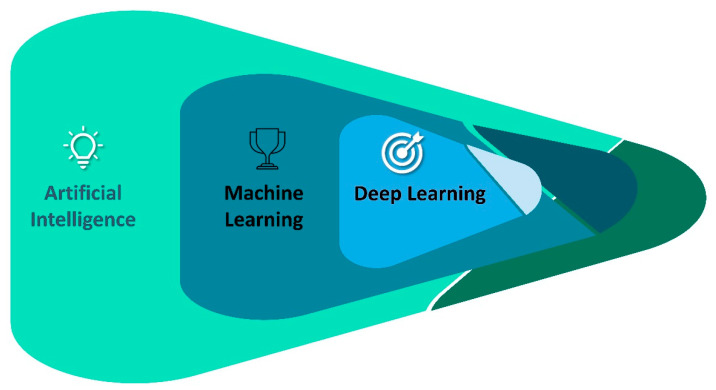
A simplified relationship of artificial intelligence fields.

**Figure 3:**
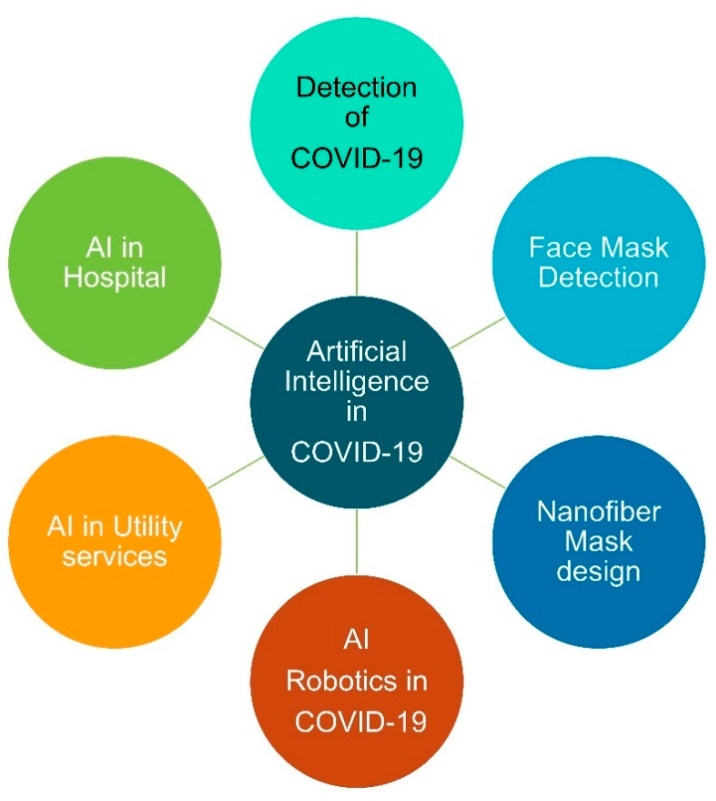
AI and its applications in various healthcare fields discussed in this review.

Machine learning (ML) is a subset of applied AI that automatically assimilates patterns and assists in decision-making without much human intervention. In ML, machines learn from a particular set of data or patterns and accomplish tasks based on the learned model with limited human intervention, rather than normal computer programs generated based on human capability and knowledge [33,34]. Deep learning (DL), a subset of ML algorithms consisting of different layers of neural networks, has seen tremendous success in the last decade, driven by large datasets (Figure 2). DL shows a rapid advancement in data processing and learning from text, speech, and images [35,36]. ML and DL are highly useful in medical image analysis [37], cardiology [38], brain stimulation [39], and cancer treatment [40]. In the fight against the COVID-19 pandemic, AI is widely accepted in many sectors of our society, including hospitals, face mask detection technology, nanofiber mask design, robotics, utility services, and so on (Figure 3). In this review, we aim to address the multidisciplinary role of AI and its subfamily involvement in healthcare to curb the pandemic effect and current challenges. Every section discusses the current scenario, the AI tools implemented in recent times, and their applications. Section 2 details the Materials and Methods section. Section 3 discusses the Omicron variant and its effect via AI tools; secondary infections such as (i) mucormycosis, (ii) blood coagulation, (iii) blood stream infection and urinary tract infection (UTI), and (iv) antibiotic resistance; AI in X-ray and CT scan image analysis; a face mask detection system by AI; and AI- and ML-based nanofiber mask and respirator designs. Section 4 discusses the use of AI-programmed robotics in healthcare, the COVID-19 pandemic’s impact on public life, AI-based decision-making, telemonitoring of patients, and AI laws and regulations in various countries.

## 2. Materials and Methods

### 2.1. Search Strategy

This review followed the Preferred Reporting Items for Systematic Reviews and Meta-Analyses (PRISMA) for article selection. We performed a search of articles with the keywords “artificial intelligence”, “corona virus”, “nanofiber materials used in healthcare”, “facemask detection”, and “Omicron”-related work in Google Scholar, Web of Science, PubMed, and Science Direct libraries with the duration between 2018 and 2022. All searches were completed in October 2022. We included original research papers and reviews published in the past 5 years until 2023 that focused on our objective. The selected servers generated a large volume of article information, such as Google Scholar (8500), PubMed (5054), Web of Science (2830), and Science Direct (790). The results were sorted carefully based on our objectives [41]. Additional studies were identified through manual searching of specific websites and bibliographic citation searching. Two reviewers (A.B. and V.M.) independently assessed the eligibility of all identified titles and abstracts of extracted articles using a set of inclusion and exclusion criteria. The doubtful study materials were chosen, and their authors were contacted for additional information to clarify their suitability before being finally included in the study. All findings were compared in research meetings with the research team to avoid discrepancies, and if any did exist, the research team resolved them (B.K., R.M., and R.S.).

### 2.2. Study Selection

**Figure 4:**
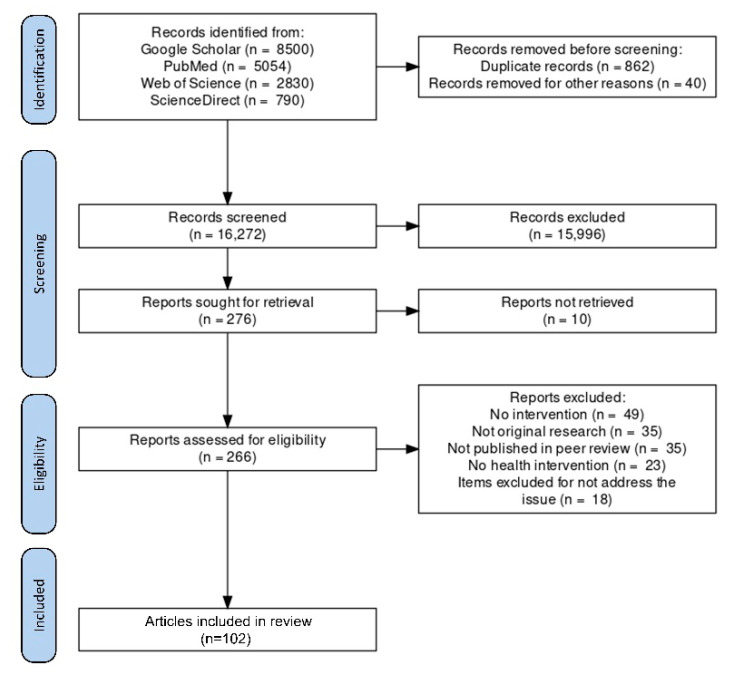
A PRISMA flow diagram for the article selection process is included in this review.

The search strategy applied in various databases yielded 17,174 articles. Duplicates were screened and removed; a total of 16,272 titles and abstracts were screened, with 276 selected for inclusion. Of those, 164 articles were excluded for the reasons mentioned below: no intervention (n = 49), not original research (n = 35), not published in a peer review (n = 35), no health intervention (n = 23), and item excluded for not addressing the issue (n = 18). Finally, 102 articles were included in this review that fall under its scope. The above selection process is outlined in Figure 4.

## 3. Omicron

Since the outbreak of the pandemic, various strains of the coronavirus have been reported around the world. However, the recent findings of variant B.1.1.529 reported it as a Variant of Concern (VOC), labeled as Omicron, from Africa in November 2021. It has reached several countries. A high number of mutations were observed in the spike protein, which was more highly transmissible among populations than the previous known variants of coronavirus [42,43]. On 11 November 2021, Botswana reported a novel SARS-CoV-2 strain, B.1.1.529, known as Omicron, a variant of the concern strain, followed by South Africa on 14 November 2021 [43,44,45]. At the beginning of January 2022, a new variant, BA.2, a subvariant of Omicron B.1.1.529, instigated a new wave of COVID-19 cases in various countries [46]. A Danish study found that BA.2 re-infects recovered patients and the vaccinated population in a shorter time frame with increased hospitalization [47]. Later, on 19 January 2022, the UK Health Security Agency identified the subvariant XE, which is a recombinant of BA.1 and BA.2 Omicron. A warning was raised by the WHO against XE, which could be more transmissible than other variants [48]. The identification of BA.4 and BA.5 variants from South Africa saw a surge in cases up to 50% in the beginning of April 2022 [49]. The recent rise of Omicron cases in various parts of the world raises concern, as this variant is identified as being highly transmissible and infectious in vaccinated populations. This demonstrates the variant evading the immunity generated by nature in the body as well as the vaccine administered recently [45,50]. The SARS-CoV-2 virus spike glycoproteins infect humans by interacting with the angiotensin-converting enzyme 2 (ACE2) receptor for connection and infection. Therefore, the vast majority of the emergency-approved vaccines and medicines are targeting the interaction of the virus spike protein and ACE2. Thus, understanding the Omicron interaction hotspot is crucial in developing successful therapeutics and vaccine upgrades [51]. The Omicron variant consists of 15 mutations in the receptor-binding domain (RDB), with 30 amino acid replacements in the spike glycoprotein, including 3 deletions and 1 small insertion [50]. AI plays a vital role in combating coronavirus infections: a well-trained AI model in various SARS-CoV-2 datasets has predicted that Omicron is 10 times more transmissible than the original Wuhan coronavirus and a couple of times more infectious than the Delta variant. Current therapeutics target the spike (S) protein receptor-binding domain (RBD) of coronavirus and angiotensin-converting enzyme 2(ACE2) of human interaction. This S-ACE2 binds together to transform the virus into the host cell and initiate infection. In this case, antibodies that strongly bind to RBD will defeat the virus. In various studies, the binding free energy (BFE) between S-RBD and ACE2 is directly related to virus infection rates [52,53]. To obtain an accurate prediction using a machine learning model, two datasets were used in this study: SKEMPI 2.0 and SARS-CoV-2. Along with this, 132 antibody–RBD complexes 3D structure were used in this study to validate the output. The BFE changes caused by mutations were predicted by the DL model in two steps: the 3D structure of protein–protein interaction (PPI) complexes and a deep learning algorithm using artificial neural networks (ANNs) built for scanning the mutations available in the TopNetmAb model. The findings state that 15 RBD mutations can play a vital role in infection, vaccine evasion, and antibody resistance. The DL model used in this study was tested with thousands of datum, so this gives a valid report as Omicron is 10 times more infectious and transmissible compared to the original coronavirus and evades vaccination with RBD mutations [54]. The overall mutations identified in the Omicron strain from South Africa are 62, but the mutations identified in the strain isolated from Australia consist of 67 mutations in all proteins. The S protein in this Australian variant has 37 mutations out of a total of 67 mutations, with 15 of the 37 mutations occurring in the RBD region [55]. This shows the Omicron variant constantly changing its structure to battle against the immune system and vaccines. A careful observation of the Omicron variant spreading across countries needs to be carried out to combat any future variants. In the meantime, COVID-19 patients acquired virus- and drug-induced immunosuppression during treatment, which created several cases of high-risk secondary infections through bacterial and fungal infections [56].

### 3.1. Secondary Infections

Secondary infections in patients are not new to healthcare, and bacterial pathogens are present in the respiratory tract of influenza virus-infected patients and are a major source of infection that causes severe illness and even death; timely diagnosis and medication are mandatory in this condition [57,58]. It was reported that 20–30% of the bacterial co-infections were associated with SARS-CoV1 and MERS-CoV coronaviruses in previous pandemics. Hospital admissions are also complicated with these co-infection patients because of the increased possibility of acquiring hospital-associated infections, which make patients more vulnerable to treatment and contribute to the surge in antibiotic intake [59]. The co-infection was considered under three distinct scenarios: (i) bacterial infection or colonization followed by SARS-CoV-2, (ii) virus- and bacteria-combined pneumonia, and (iii) secondary bacterial superinfections [60]. A detail study of possible secondary and co-infections was assessed by Lai et al. and showed bacterial infections such as *Klebsiella pneumoniae*, *Streptococcus pneumoniae*, *Acinetobacter baumannii*, *Staphylococcus aureus*, *Legionella pneumophila*, *Chlamydia pneumonia*, and *Mycoplasma pneumoniae*, and viruses such as influenza, metapneumovirus, coronavirus, rhinovirus/enterovirus, influenza B virus, HIV virus parainfluenza, and candida species and *Aspergillus flavus* [56]. Apart from microbial infections, we specifically propose to utilize the AI-based methods in four selected cases to minimize the pressure on healthcare workers in critical conditions: (i) mucormycosis infection, (ii) blood clotting, (iii) bacterial infections in the blood stream and urinary tract (UTI) in COVID-19 patients, and (iv) antimicrobial resistance.

### 3.2. Mucormycosis

**Figure 5:**
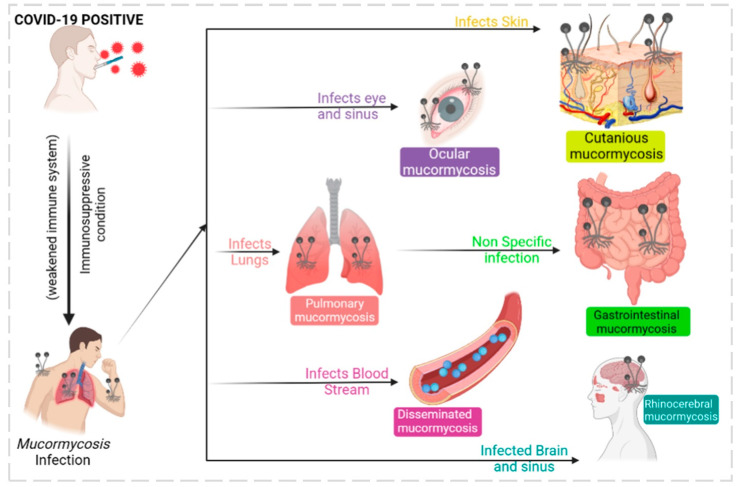
Mucormycosis infections of various types are associated with COVID-19.

**Figure 6:**
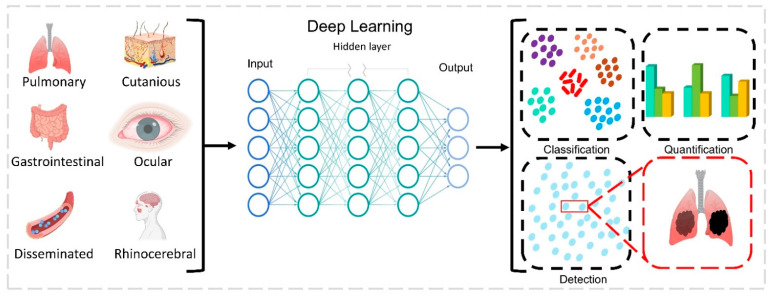
Deep learning-based imaging and detection of mucormycosis infection from images and data. Affected and standard images of rhinocerebral, ocular, cutaneous, pulmonary, and around 30 different parameters are used as input. Deep learning software is designed based on a human brain-like model, which consists of artificial neurons (nodes) organized next to each other in three different layers. (i) The input layer is provided with input data; (ii) the hidden layer executes different types of calculations, predictions, and operations; (iii) the output layer provides output data. Different knowledge from the health worker, such as diagnosis and medication; entered features such as sex, place, duration of diseases, COVID-19 symptoms, CAM symptoms; and numerical features such as age can be used by the deep learning model. Image classification, detection, and segmentation are the most common applications of deep learning model. Image classification determines whether an image is infected or not. Segmentation is a partition analysis to find infected and uninfected images.

The mucormycosis fungus infections in post-COVID-19 or secondary infections have become life-threatening invasive species; since they were associated with COVID-19, they are represented as CAM. They belong to the order *Mucorales* with different genera such as *Saksenaea, Mucor*, *Rhizopus*, *Lichtheimia*, *Cunninghamella,* and *Rhizomucor*. They are opportunistic fungus species that are prevalent in immunocompetent patients, accounting for up to 19% of all COVID-19 cases [61]. Recent findings show CAM have most of their cases in India, nearly 71% of global cases, and 140 cases per million [62]. The prevalence of CAM is due to the huge number of diabetic cases in India. Unfortunately, CAM primarily infects diabetic, diabetic ketoacidosis, and diabetic mellitus patients, accounting for 50%, 18%, and 57%, respectively. As shown in Figure 5, clinical diagnosis reports that CAM proliferates in the rhinocerebral, disseminated, pulmonary, gastrointestinal, cutaneous, and ocular regions. [63]. An earlier diagnosis of CAM opens earlier treatment possibilities and prevents numerous deaths. Currently, CT scans, MRI scans, and cell biopsy tests are used to identify CAM; however, all these analyses are expensive for middle- and lower-class people. In this context, an advanced AI model was developed to predict mucormycosis in the discharging patients of a hospital in India. The scikit-learn library was used for logistic regression, decision tree, and random forest, and an eXtreme gradient boosting algorithm from XGBoost library was utilized to analyze 1229 COVID-19-discharged patients and 214 inpatients who tested positive with mucormycosis infection. In total, 35 variables were used for the prediction model with a 5-fold cross-validation method to produce a reliable output. A competent performance was observed between logistic regression: 95.0, XGBoost: 94.0, and random forest: 94.0. The model XGBoost exhibited accuracy and precision of 0.91 ± 0.026 and 0.67 ± 0.0526 tailed by logistic regression. The AUROC value of 0.95 ± 0.023 was achieved by logistic regression, nearing a score of 1, which is a precise value. The study used five variables such as obesity, nasal discharge, myalgia, de novo diabetes, and anosima to show the possibility of mucormycosis infection [64]. Recent studies showed uncontrolled diabetes favored mucormycosis infection in post-COVID-19 patients [13,15]. Similarly, an AI-based modified deep learning algorithm strategy known as tha “hydrid learning-based neural network classifier” (HLNNC) was used to identify the CAM, with the help of popular techniques such as the convolutional neural network (CNN) and support vector machine (SVM). Real-time image records were used with image processing procedures such as (i) image acquisition, (ii) pre-processing, (iii) feature extraction, and (iv) classification, in dataset training. The HLNNC analysis produced an excellent accuracy rate of 99.5%, which was implemented through Python. Cross-validation was completed using CNN and SVM, showing 95.5 and 85.0% accuracy. Every model will have some practical cons or demerits; this model should be improved with time-constraint algorithms to obtain a better and faster output during rush hours in hospitals [65]. A detailed schematic representation of deep learning and its functions is given in Figure 6.

*Limitations*

In general, the models mentioned above have some limitations for hospitals. Adding more variables will help achieve more prominent results. Nealy one thousand discharged patient data sheets were used for the identification of mucormycosis, but the data used in this study were from only one hospital. Since India has a huge population and nearly half a million hospitals across the country, utilizing a minimum of 10% of the mucormycosis-related data from these hospitals will improve the program’s accuracy in detection. Apart from this, granular, diabetic ketoacidosis, tumors, and neutropenia details were lacking in the used model. Further, a longer time span and a larger dataset are important to achieve accurate results. This could be taken into account when determining how soon after discharge a patient might develop mucormycosis.

### 3.3. Blood Coagulation

**Figure 7:**
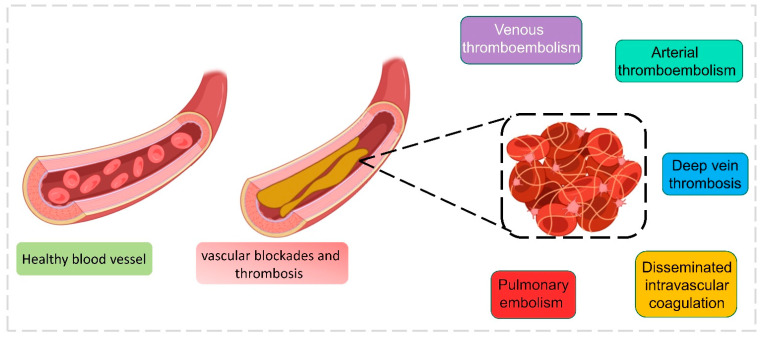
Different types of blood coagulation during the COVID-19 infection.

Blood coagulation or clotting occurs during cellular/tissue injury externally to protect against loss of body fluids. Coagulation disorders are frequent in COVID-19 patients, which exhibit the severity of the disease. The inflamatory response that occurred post-infection displayed a discrepancy in coagulant and anticoagulant mechanisms mediated by endothelial dysfunction [66]. Reports illustrated severe COVID-19-infected patients expressed reduced antithrombin levels and elevated fibrin degradation products, fibrin and D-dimer. COVID-19 patients admitted in the ICU were administered with low molecular heparin (LMWH) to avert the progress of thrombosis which may result in thromboembolism or stroke [67]. Patients with COVID-19 and coagulation factors had higher mortality than normal patients [68]. The major types of reported coagulation complications are (i) arterial thromboembolism, (ii) deep vein thrombosis, (iii) disseminated intravascular coagulation, (iv) pulmonary embolism, and (v) venous thromboembolism [69,70,71,72] (Figure 7). Patients cannot be diagnosed with venous thromboembolism symptoms; it is found mostly with a sudden pulmonary embolism. Observations made in a Chinese hospital with 81 patients showed an elevated level of venous thromboembolism, which was directly proportional to an increase in the d-dimer level, and this level was reduced when the patient was treated with anticoagulant therapies; moreover, COVID-19 patients who have gone through a ventilator are more prone to venous thromboembolism complications [73]. Deep vein thrombosis is reported more often in COVID-19 patients, where the clotting occurs deep inside the veins. Similarly, as with venous thromboembolism, d-dimer was highly associated with deep vein thrombosis and can be used as a diagnostic marker, which was observed from the data of 1783 patients directly related to their ICU duration [74]. Arterial thromboembolism is the blockage of an artery that can reach distant organs in COVID-19 patients. This event can even cause limb loss and death under certain conditions. It happens due to the extensive use of thromboprophylaxis, but when patients aged under 60 are treated properly, the mortality can be reduced [69,75]. Pulmonary embolism occurs in numerous COVID-19 patients, with a high mortality risk. D-dimer can be used as an effective marker to identify pulmonary embolism, since the level is higher in patients with pulmonary embolism [76]. Coagulation of blood occurs in COVID-19 patients even after several weeks of recovery, since with a huge number of new cases, regular follow-up is not possible with patients, so it is considered as prognostic factor [77]. In critical situations, patients’ conditions are carefully monitored through coagulation indicators such as fibrogen degradation products, d-dimer, lymphocytes, and accumulated platelets, which are more helpful in doctors’ decision-making [78]. Specific laboratory analyses such as thrombin time (TT), prothrombin time (PT), fibrinogen (Fbg), D-dimer (DD), and activated partial thromboplastin time (aPTT) are analyzed to predict the coagulation condition in patients [78]. To reduce the burden on laboratory analysis, ML algorithms are more useful to predict, identify, and differentiate results. The authors Fang et al. developed ML algorithms to identify the clots in samples from laboratory test results. A training dataset was formed with 192 clot-identified samples and 2889 non-clot-identified samples retrieved from the laboratory information system. This dataset was trained with a backpropagation neural network (BPNN), which has the ability to “learn” itself from the given data and adjust the parameters by itself, and it was barely trained with the five-fold cross-validation method. The output consisted of five values, inclusive of a PTT, PT, Fbg, TT, and D-dimer. The D-dimer level was also concentrated due to its high presence in clots. The area under the ROC curve accuracy was 0.906, and the classification accuracy was 0.829 after analysis. Since BPNN was trained with five different variables, it could easily detect clots in samples by analyzing the coagulation results from the laboratory database and making the decision-making process easy for the technician. The advantage of BPNN is that it can be embedded directly in an instrument program and used without much difficulty [79]. Similarly, Kremers et al. have developed an ML-based neural network to predict thrombosis in COVID-19 patients. Two sets of patient data were used for this study: set 1 consisted of 133 COVID-19 patients admitted under suspicion of infection, and set 2 consisted of 16 severe patients admitted to the ICU. The neural network model predicted the thrombosis by analyzing the patient’s data during admission along with the laboratory results. A higher thrombin generation (TG) level was observed in COVID-19 patients who suffered from thrombosis, which was taken into account. Around 33 variables were used to train the neural network model that predicted thrombosis. A 98% prediction success rate was observed in general admission, and a 100% successful prediction in ICU admission was successfully obtained [16].

*Limitations*

This neural network method has the ability to analyze limited samples, and different parameters or variables are not immediately available from regular clinical laboratories. The accuracy rate was reduced if we did not include thrombin generation and thrombin dynamics parameters.

### 3.4. Bacterial Infections in the Blood Stream and Urinary Samples of COVID-19 Patients

#### 3.4.1. Blood Stream

The bacterial co-infection occurs mostly during the seasonal viral respiratory track infection, which is directly related, and increases the risk of patient health conditions. Previous influenza pandemics revealed that mortality increased due to bacterial co-infections. Recent research suggests that *Acinetobacter baumannii* co-infection exists in COVID-19 patients admitted to the ICU [80]. In the earlier months of the 2020 year, a confirmed bacterial co-infection with laboratory output was found to be 7%. Further, patients admitted to the ICU showed a higher prevalence than normal patients, which was obtained by screening various databases and datasets [80]. Various bacteria were identified as co-infectants, but some were remarkable in increasing the severity of patients, such as *Streptococcus pneumoniae*, *Acinotobacter baumannii*, *Legionella pneumophila*, and *Klebsiella pneumoniae* [81,82]. The biological question of whether a failure of antibiotics or antibiotic resistance occurred in the co-infection bacterial community needs to be considered. Bloodstream infections (BSI) are an emerging cause of death and illness in COVID-19-infected patients; it is difficult to identify secondary bacterial infections during hospitalization, which should be predicted earlier to begin antibiotics and save severe COVID-19 patients. Secondary bloodstream bacterial infections were reported more frequently in recent times, from 14.3 to 67.7% of acute COVID-19 patients admitted to the ICU [83,84]. This may lead to prolonged hospitalization and mortality in certain cases of bacterial superinfections [85]. Martinez-Guerra et al. from Mexico studied 69 patients with COVID-19 for healthcare-related pneumonia in hospitalized patients, with *Enterobacteriaceae* accounting for 69.6%, non-fermenting *Bacilli* for 26.1%, and *Staphylococcus* for 40% of the bloodstream [86]. Contou and collaborators conducted a study with 92 patients admitted to the ICU with acute respiratory failure. During ICU admission, 26 (28%) of them were co-infected with a pathogenic bacterium, and 32 bacterial pathogens were identified through laboratory procedures. Of the 32 bacteria: (31%) methicillin-sensitive *Staphylococcus aureus*, (22%) *Haemophilus influenzae*, (19%) *Streptococcus pneumoniae*, (16%) *Enterobacteriaceae*, (6%) *Enterobacteriaceae*, (3%) *Moraxella catarrhalis*, and (3%) *Acinetobacter baumannii*. Further analysis revealed that seven bacterial isolates were resistant to third-generation cephalosporins and amoxicillin-clavulanate combinations [87]. According to a recent study, critically ill COVID-19 patients from west India were found to be infected with BSI. Among 750 patients admitted to the COVID-19 ICU, 64 (8.5%) developed BSI and became critically ill. Of those, 53 (82.8%) patients were infected with a Gram-negative pathogen. *Acinetobacter baumannii* was found to be prevalent, along with *Klebsiella pneumonia*. Furthermore, multidrug-resistant organisms were found in approximately 57.8% of *K. pneumoniae* and *Enterococcus* members; interestingly, carbapenem-resistant Gram-negative bacteria were found in approximately 47.2 percent of cases. Among these was *Acinetobacter baumannii*: an opportunistic pathogen that can affect immunocompromised patients and was possibly acquired from the ICU. The overall impact of BSI encouraged multiple disease conditions with increased leukocyte, mechanical ventilation, and multiple organ failure, which should alert the clinicians for BSI [18]. The ICU-acquired BSI was high in patients who stayed for a longer duration. The diagnosis of BSI is difficult in patients treated with tocilizumab because it is immunosuppressive, so the identification of BSI symptoms such as fever cannot be detected [84]. Another study was conducted by Pourajam et al. in an academic medical center in Isfahan, located in central Iran. COVID-19 patients admitted in two ICUs were taken into account for BSI identification, and an antimicrobial susceptibility test was conducted to identify bacterial infections among 553 patients admitted for COVID-19 and severe pneumonia in the ICU. There were 65 (11.9%) patients with secondary bacterial infections, with a median age of 69.4 years (ranging from 21 to 95). Particularly, n = 65 (100%) patients treated with antibiotics reported positive results for bacterial culture, with meropenem for 12 days and levofloxacin for 2–24 days. *Klebsiella pneumoniae *and *Acinetobacter baumannii* were reported to be the most prevalent secondary bacterial infections in (n = 44) and (n = 33) persons present in blood samples. The secondary bacterial infection in patients showed a broad range of drug resistance and increased mortality up to 83% against a total mortality of 38.1% in the overall admitted population of 553. The prevalence of Gram-negative bacilli resistance to carbapenem was observed in ICU patients with high proportions of *K. pneumoniae* and *A. baumannii* [88]. Palanisamy et al.’s previous study discussed the presence of carbapenem-resistant bacteria from India. The identification of BSI through ML is more interesting and is a great help in clinical diagnostics. Pai et al. developed a prediction model to identify the BSI in patients. A total of 4275 patients’ records were accessed to identify BSI at a general hospital in Taiwan from 2015 to 2019. A total of 12,090 blood culture reports were taken and scrutinized, totaling 5075, with 1478 BSI infections and 3597 non-BSI. Five ML models were included: support vector machine (SVM), XGBoost, logistic regression (LR), random forest (RF), and multilayer perceptron (MLP). Nearly 30 different variables were used to analyze the BSI presence. The threshold of 0.5 was fixed to determine the presence of BSI, and if the model exceeded the value, it was determined the sample was infected with BSI. At 0.724 and 0.706, XGBoost had the highest sensitivity when validating the datasets. Further, SVM (0.578 and 0.566), RF (0.565 and 0.577), and MLP (0.494 and 0.406) models exhibited lower sensitivity. The validation was conducted with the important risk factors associated with BSI, such as prothrombin time (PT), platelets (PLT), and albumin (ALB), which are key factors [89]. Similarly, Zoabi et al., developed a patient outcome of BSI model to identify BSI patient risk based on the electronic medical records (EMR) of only bacteria-based positive blood culture results, with the goal of identifying risky patients, initiating a planned treatment with appropriate antibiotics, and transferring them to the ICU. The poor outcomes of hospitalized patients were concentrated to minimize the mortality rate. An area under receiver operating characteristics curve (AUROC) of 0.82 was achieved for the inclusive prediction model, and 0.81 was achieved for the compact model with 25 limited features. The poor outcome of the patient was predicted by the influences of low albumin, high creatinine, and high red cell distribution width. Albumin and high red cell distribution width are associated with patient mortality and used as prognostic markers in various studies. The main objective of this model was to identify the poor outcomes of patients receiving hospital treatment for the first time. This model was developed based on the data collected from EMR between 2014 and 2020 [90]. Numerous studies were conducted to identify patients related to sepsis-like infections, but BSI-based research models are very limited, and this is a research gap that needs to be considered to develop more models that could be directly helpful for healthcare during pandemic situations, since BSI is directly connected to COVID-19 patients.

*Limitations*

The above-mentioned model has its own demerits. Since this model is based on EMR data, patient influence on the EMR or partiality could reflect on the patient’s treatment. This model could be used for confirmed BSI-positive cases but cannot be used for general identification of BSI since this model was developed to alert healthcare professionals about the risk condition of BSI patients. Earlier blood culture results are used to identify the BSI patient, but final microbiological results should be added to determine the complete treatment conditions.

#### 3.4.2. Urinary Samples

Urinary tract infections (UTI) are very common bacterial infections, infecting 150 million people every year. UTIs refer to bacterial or pathogenic infections that occur in the urinary system and include urethritis, renal abscess, pyelonephritis, prostatitis, and cystitis. UTI infections occur most commonly with Gram-negative and Gram-positive bacteria, along with rare fungal infections. Uropathogenic *Escherichia coli* (UPEC) has been linked to both common and severe illnesses in patients [91]. Apart from UPEC infection, various bacteria result in UTIs, including *Klebsiella pneumoniae*, *Proteus mirabilis*, *Pseudomonas aeruginosa*, *Enterococcus faecalis*, *Enterobacter cloacae*, *Streptococcus bovis*, and the fungus *Candida albicans* [92,93]. During the pandemic, to avoid hospitalization and prevent COVID-19 infections, patients were treated with broad spectrum antibiotics for UTI-related infections. It was reported that overall antibiotic usage was higher in COVID-19 patients, but the bacterial co-infection percentage was very low [94]. The majority of co-infection studies concentrated on pulmonary and related co-infections, but UTI co-infections in COVID-19 patients have been reviewed very little. A recent study stated that, among 140 patients admitted in the ICU, only 8% reported to have UTIs, and mostly catheter-associated UTIs [95]. Similarly, 3% of patients were identified with UTIs of a total of 1016 patients admitted to five hospitals in the USA [96]. Díaz Pollán et al. made a study of the UTI and catheter-associated urinary tract infections (CAUTIs) superinfection group, with a total of 87 COVID-19 patients admitted to the ICU from 2281 patients in a hospital in Spain. Of that, 10.3% were community-acquired UTIs and 89.6% were hospital-acquired UTIs known as superinfections. The recovery of UTI patients was greater than the superinfection group’s patients in the ICU, and the mortality of superinfection was recorded as 26.4%. A prolonged stay in the ICU for up to 27 days led to hospital-acquired bacterial infections, which turned into superinfection in patients. A bacterial analysis of urine culture revealed 95 isolates, the most common of which was *E.coli*, followed by *Enterococci* (*E. faecalis* and *E. faecium*), and *Pseudomonas aeruginosa*. These bacteria expressed resistance against quinolones and B-lactams; further, Gram-negative bacilli producing carbapenemase were prevalently found in UTI superinfection patients, and a higher leukocyte count was noted in these patients [22]. It was previously reported that *Enterococci* play a crucial role in UTI infections [95]. An increased level of BSI incidents was noted with patients infected with an elevated level of *Enterococcus* [97]. When dealing with large datasets in the thousands, UTI record analysis and diagnosis are difficult. Taylor et al. analyzed five AI algorithms to find the best model that can be useful in handling huge volumes of files with the highest specificity and sensitivity in UTI diagnostics. In total, 80,387 files of urine culture results from the emergency departments of four hospitals, which maintained a centralized data storage system, were used to identify the best model. Six models were developed with the ML algorithm for UTI prediction: random forest, adaptive boosting, SVM, extreme gradient boosting, elastic net, and neural network. Two sets of models were developed, with 211 variables used for the full set under eight headings (urinalysis, physical findings, demographics/arrival information, vitals, labs, past medical history, and outpatient medications) and 10 variables used for the reduced set (age, gender, UA leukocytes, UA nitrites, UA bacteria, UA blood, history of UTI, dysuria, UA WBC, and UA epithelial cells). UTI predictions were compared between six models, and XGBoost was found to give the most accurate report based on an area under the curve (AUC) of 0.904 for full models and AUC of 0.877 for the reduced model. Therefore, XGBoost expressed higher sensitivity with the UTI diagnosis in a huge volume of HER datasets in diagnosing urine culture results than the previously reported studies [98]. Similarly, handling a huge volume of laboratory analysis is a complicated situation that creates a state of exhaustion in laboratory technicians. A classification of samples completed before the analysis will reduce a huge work burden and save the economy during the pandemic situation. Most of the analyses in the laboratory were conducted due to suspected infections in UTIs, where two-thirds of the urine culture results were negative. Screening criteria are much more helpful to detect and use the most accurate bacterially infected samples for analysis. Data of urine samples prior to culture were analyzed from a microbiology laboratory connecting three hospitals from the United Kingdom. Reports of urine microscopy, culture, and sensitivity reports were used to compare two classification models: (i) the heuristic model with the grouping of white blood cell count and bacteria count, and the (ii) ML approach, to test algorithms of random forest, neural network, and extreme gradient boosting with different variables. A total of 2,12,554 patient urine reports were analyzed: the ML algorithm outperformed the heuristic model and exhibited an improved output to reduce the workload in the laboratory sample processing up to 95% sensitivity. The sample analysis of pregnant women and children under 11 years was highly important. Since then, an independent XGBoost algorithm was trained separately for this purpose and achieved 41% reduction in the workload of urine culturing and 95% sensitivity in detecting UTI-positive samples among each group of pregnant patients, children under 11 years, and general patients [99]. A clinical analysis of urinary infection-based biomarkers is very important in identifying UTIs: these biomarkers are key variables in validating the ML-based algorithms, so choosing the right variables will be helpful in obtaining a more precise value. CAUTI and UTI become high risk factors when associated with hospital-acquired infections, and if untreated early, they lead to mortality in COVID-19 patients [21]. AI and ML models are highly helpful during this COVID-19 pandemic situation to minimize the workload of healthcare workers. To reduce the workload, similar algorithms are recommended for use in hospital and laboratory settings.

### 3.5. Antibiotic Resistance: Improving Diagnosis Using ML

The forceful management methods carried out during the COVID-19 pandemic brought down many seasonal infections such as influenza, pneumococcal diseases, and tuberculosis compared to previous years, as an added benefit [100]. Many healthcare institutions and public communities used antibiotics as a therapy/self-medication to prevent COVID-19 infections without proper exposure to their effects, which increased antibiotic resistance (AR) [23,24]. Getahun et al. stated that 72% of COVID-19 patients were treated with antibiotics, whereas only 8% of the patients were infected with bacterial and fungal co-infections [23]. Easy access to antibiotics without a proper prescription in lower- and middle-income countries, frequent intake, and uncontrolled distribution are the primary reasons for AR. Reports stated that 69% of COVID-19 patients took antibiotics during treament, namely, ceftriaxone, azithromycin, moxifloxacin [101]. The *Carbapenem-Resistant Enterobacteriacea* was identified in the ICU of a hospital in Italy during the period of 2019–2020, where the ICU was allotted for COVID-19 patients. It was reported that patients were infected with hospital-acquisition infection and colonization of this bacteria, and acquisition went from 6.7% in 2019 to 50% in 2020 [102]. Among 102 hospitalized patients in Wuhan, China, 159 bacterial strains were isolated: *Acinetobacter baumannii* was the predominant strain found in patients (n = 57), followed by *Klebsiella pneumoniae* (n = 49) and *Stenotrophomonas maltophilia* (n = 10). Carbapenem resistance was found to be 91.2% and 75.5% in this study [25]. Another finding from a hospital in Italy stated that the co-infection of bacteria in patients admitted to the COVID-19 ward was identified with *Mycobacterium tuberculosis*, *S. aureus*, *Klebsiella* spp., *Mycoplasma pneumonia*, *Haemophilus* spp., *S. pneumoniae*, and *Legionella pneumophila.* Co-infection was reported to be higher in the patients admitted to the ICU with AR, than in the general ward. In this situation, patients’ regular use of broad-spectrum antibiotics must be reconsidered, and the Antimicrobial Stewardship Program guidelines must be followed [103]. AI-based applications are more helpful in combating the AR conditions. The ML was used as a clinical decision support tool in various studies for the prediction of antimicrobial resistance (AMR) and AR in healthcare [104,105,106]. An automated ML was used to identify the antibiotic susceptibility using an antimicrobial susceptibility dataset of 11,496 acquired from 499 patients admitted to a public hospital in Greece. A stack ensemble algorithm present by default in Microsoft Azure AutoML was used for the analysis, along with the VoltingEnsemble, MaxAbsScaler, LightGBM, SparseNormalizer, and XGBoostClassifier algorithms, which were used together for a better tuning process for the antibiotic susceptibility prediction. Various attributes were used from the dataset, such as sex, age, sample type, Gram stain, 44 antimicrobial substances, and past antibiotic susceptibility reports. The stack ensemble algorithm was found to be best fitted with an area under the curve weighted (AUCW) value of 0.822 and 0.850. This Microsoft Azure was used in two medicine departments as a decision-making tool by physicians [107]. Usually, this type of analysis will take around 24 h, but enhanced ML algorithms are more precise in making decisions under critical circumstances. Similarly, Feretzakis et al. analyzed a method to help in predicting the AMR of *Acinetobacter baumannii, Klebsiella pneumoniae*, and *Pseudomonas aeruginosa*, mostly reported from the ICU and wards of hospitals. As mentioned above, it will take about 24 h for the results of general antibiotic tests. However, in this study, five ML models (JRip, RandomForst, MLP, Class.Regr, REPTree) were used to predict the AMR, with the data of hospitalized ICU patients received from the microbiology laboratory of a Greek public hospital. The dataset included various attributes such as age, sex, gender, bacterial species, and samples from blood, urine, pus, and tissue were collected. The classification via regression model produced an ROC area of (0.933 and 0.918), and the training was given based on the antibiotic susceptibility pattern for Gram-negative bacteria with the highest resistance rate, which were most prevalent in the ICU [105]. There was a similar study undertaken by Wang et al. to identify the antimicrobial phenotype resistance from the genome data of *Staphylococcus aureus* using ML. The whole genome data were used to identify the minimum inhibitory concentration of 466 *S. aureus* isolates using the k-mer algorithm, along with three combined ML algorithms: random forest, SVM, and XGBoost. For the MIC analysis, nearly 10 antibiotics were chosen, including clindamycin, cefoxitin, oxacillin, levofloxacin, trimethoprim-sulfamethoxazole, vancomycin, linezolid, erythromycin, daptomycin, and gentamicin. The k-mer algorithm sucessfully predicted the cefoxitin resistance, concluding that the model can identify methicillin-resistant *S. aureus* with accuracy >91% to 93% [108]. The sequence data of nearly 500 isolates were used in this study; training under large datasets will improve the algorithm to have greater accuracy. Patients are prescribed antibiotics based on the susceptibility results, but this algorithm can be used in the healthcare setup where there are limited resources to obtain antimicrobial susceptibility tests rapidly. There are limited studies available related to ML-based screening; some are mentioned in Table 1 below.

*Limitations*

There are very limited research outputs in the ML-related screening of antibiotic resistance and antibiotic susceptibility in hospital-based datasets, since many large healthcare setups have a huge dataset that can be used to screen for a lot of antibiotic resistance bacteria. However, receiving those datasets for research purposes is not an easy task, which is a major setback.

### 3.6. AI Detection of COVID-19 and Preventive Measures with AI

**Figure 8:**
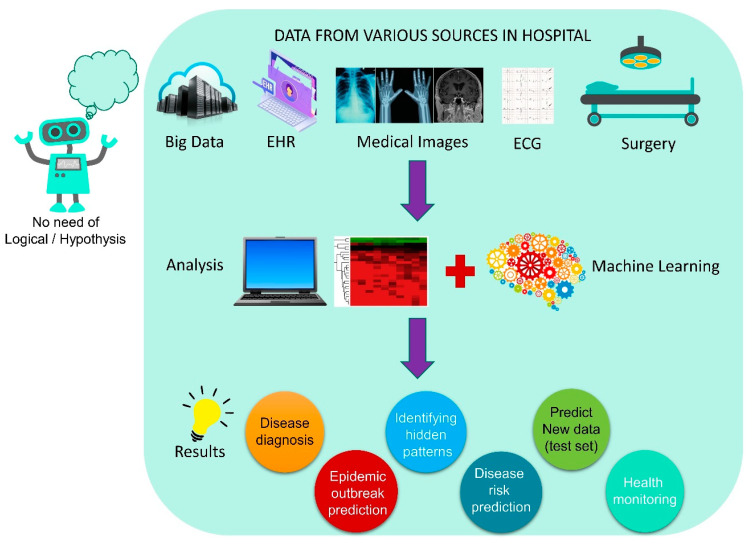
Various applications of ML driven from hospital data.

AI-based approaches were widely used in the recent COVID-19 pandemic diagnoses. The daily infection rate and the survival ability of patients in South Korea were analyzed using machine learning and deep neural networks with logistic regression and support vector machine (SVM) [113]. The radiographic images play an important role in distinguishing coronavirus infection and other respiratory lung infections such as seasonal flu, tuberculosis, etc. The various applications of AI and machine learning are detailed in Figure 8. The recent study by Harmon S.A et al. in developing a lung segment model for a chest CT scan to detect the coronavirus infections, was further trained using the AH-Net architecture. Along with this, an image classification model developed as a hybrid 3D and full 3D model based on Densnet-121 architecture was considered to have a high accuracy rate of up to 90.8% [114]. Recently, the lung ultrasound (LUS) imaging technique was highly recommended in the detection of COVID-19 in lungs. When this scanning is used in the right way, there is a chance of reducing infections among health workers and patients [115]. Born et al. proposed a POCOVID-net DL-based model that was based on the VGG16 network [116]. The author employed DL architecture such as VGG16 and VGG19 [117], Inception V3 [118], ResNet50 [119], and Xception [120] as supporting tools in computer-based image analyses in screening lung ultrasound (LUS). A similar study was conducted by Zokaeinikoo et al. in analyzing the CT and X-ray with an AI model for the detection of COVID-19 (AIDCOV), which can differentiate the infected/non-infected persons along with other respiratory tract infections. AIDCOV was subjected to screen chest X-ray images and identified 475 COVID-19 cases from 3949 samples belonging to various viral/bacterial infections and 1583 normal samples from 10 balanced and unbalanced publicly available datasets. A promising output showed a 99.8% sensitivity with 100% specificity and a 99.8% F1 score for identifying COVID-19 in X-ray images, and a mean cross-validation accuracy of 98.8% and a sensitivity of 99.4% were obtained by screening a dataset of CT images [121]. This dataset held samples from around the world; therefore, interpreting samples between different people shows more promise. After facing waves of COVID-19 infections around the world, our health system and financial sectors have started collapsing. The need of the hour is a rapid technique that is a more cost-effective, easily accessible, and more accurate testing tool to identify infections and control spreading. RT-PCR techniques have some drawbacks as they detect viral RNA once the patient develops symptoms, but CT can detect it even before symptoms occur in patients [121]. CT images prove to detect COVID-19 patients, even when they are identified as asymptomatic with negative RT-PCR results [122].

### 3.7. Face Mask Detection Systems Using AI

The pandemic forced us to develop face mask detection systems, which are a new initiative in controlling the coronavirus spread among people in public places and closed environments. Thanks to the recent upgrades in face detection systems, a kind of AI technology has entered our daily lives [123,124], which has become a key platform in designing the tools for mask detection. The challenging part of mask detection is image processing. Due to different types of masks, camera resolutions, and pixels, various angles of observation, different views, obstructions, detection accuracy, and real-time detection are key functions that need to be considered. The AI consists of a subset called machine learning, which learns from the given dataset, and develops its knowledge from the dataset. DL models are represented in considerable recent research in face mask detection due to their multilayer exploiting capacity and drawing inspiration from biological neurons [125]. The DL models such as convolutional neural network (CNN) [126], ResNet-50 [127], You Only Look Once (YOLO) versions (such as YOLOv1, YOLOv2, and YOLOv3) [127,128,129,130], and mobile network (MobileNet v1 and v2) [131], showed promising outputs in identifying face mask detection. Sethi et al. (2021) proposed a model consisting of one-stage and two-stage detectors to identify objects within a rapid time and high accuracy. They used ResNet50, MobileNet, and AlexNet, the three standard baseline models in mask detection, along with a bounding box prediction method. In this ResNet50 model, which showed higher accuracy up to 98.2% in face mask detection, this model was proposed to be used for video surveillance devices [132]. Bhuiyan et al. (2020) proposed a model for identifying masked and unmasked faces using the YOLOv3 advanced version. YOLOv3 used the CNN algorithm for detecting faces. YOLO detected images by creating a linkage with CNN by using hidden layers, researching them, and retrieving an algorithm. The analysis, which was performed on 30 similar images from the dataset, yielded promising detection results. Even with a video analysis, the model worked well with frames per second inside a live video; that is, average fps was 17, which is highly promising [129]. A similar study was carried out to develop an Internet of Things (IoT)-enabled smart door capable of monitoring human body temperature and detecting face masks. In a non-contact approach, an infrared temperature sensor was used to detect the body temperature. The face mask detection system was created using the TensorFlow software library, which is open-source and free. A Pi cam was used to monitor face recognition, and a detector in the door counted the number of people inside the room, prohibited entry of excess people when a pre-defined room limit was reached, and locked the door if a person came without a mask. The Raspberry pi was used as a real-time DL system to detect a face mask and temperature. Further, the model gave 97% accuracy when it was trained using various face recognitions. This system can be used in airports, hospitals, shopping malls, offices, colleges, and so on [133]. The study by Teboulbi et al. (2021) proposed a face mask and social distancing detection model that is an embedded vision system. The author used pre-trained models such as MobileNet, ResNet Classifier, and VGG for this study. This model detected face mask violations and social distancing. The output in real time is promising, as the F1-score, sensitivity, and specificity showed 99%, along with 100% accuracy. In a real-time scenario, the detection system will raise an alarm for violating face mask and social distancing in public places. This can be used with the existing cameras in all public places for the prevention of COVID-19 infections. Similar strategies using ML algorithms have started coming to the commercial market as the coronavirus cases surge worldwide [134]. Various tools or DL models published in recent times show a promising future in face masks or facial detection technologies; recent findings are mentioned in Table 2.

### 3.8. Nanofiber Masks Using AI

#### 3.8.1. Machine Learning in Mask Production

The face mask has become a shield in fighting the coronavirus, and the adaptation of recent technology in upgrading the mask is highly welcomed due to the current pandemic situation. The coronavirus family consists of SARS-CoV, MERS-CoV, and SARS-CoV-2, which are positively charged single-strand RNA viruses. The SARS-CoV-2 virus size ranges from 50 to 200 nm in diameter with a single-strand RNA genome [142]. The machine learning is used to predict the fabric properties used to make masks and to monitor their performance. The Egyptian cotton (EC) fabric was prepared with diverse thread counts to create a three-layer stacking mask. The particle filtration efficiency (PFE) and bacterial filtration efficiency (BFE) were analyzed to identify a better combination of EC fabric masks. The thicknesses of EC fabrics were 0.182 mm for EC100, 0.128 mm for EC 200 mm, and 0.120 mm for EC300. A Porometer analysis exhibited 64.9, 79.2, and 43.5 µm from EC100, EC200, and EC300 fabrics. Different combinations of three EC fabrics in different orders also brought remarkable filtration capacities, as 1-3-1 was found to be the best combination in PFE and BFE with 45.4% and 98.1% filtration capacities, respectively. Machine learning models, Lasso and XGBoost, were used to predict the best combinations of masks in ΔP, PFE, and BFE combinations. The Lasso and XGBoost predictions were highly reliable in BFE, which was very similar with both models and material knowledge. The models were successful, even with limited training data, in predicting all three properties. ML algorithms are highly desirable as they are economical and improve experiment results in mask design [143]. The respirator burdens the wearer’s breathing ability, which leads to a decline in physical activity. To overcome this issue, machine intelligence algorithms (ML) were combined with a dynamic air filter (DAF), which is a stretchable elastomer fiber. For the first time, an air quality-responsive algorithm (AQA) and a breathing demand-responsive algorithm (BDA) were used in the filter. The AQA algorithm initiated the super flow of air under a clear atmosphere, and the BDA algorithm observed the wearer’s breathing demand and adjusted the flow rate for physical activity. This was achieved by the stretching and relaxation ability of the elastomer [144]. Since this is a pioneer study in merging elastomeric filters with ML algorithms for respirators, it can be used for growing air pollution and minimizing the pandemic effect.

#### 3.8.2. Electrospun Nanofiber Mask by Nanotechnology

**Figure 9:**
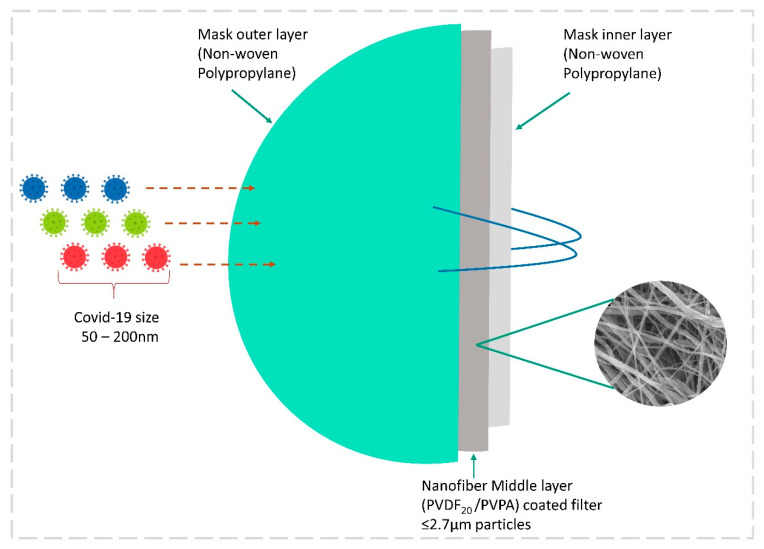
Illustration of a nanofiber mask with 3 layers of protection against micro-aerosol droplets with coronavirus.

Another major breakthrough in controlling the airborne transmission of SARS-CoV-2 is through the advanced electrospun nanofibrous air filters adopted in the mask. Electrospun nanofibers are not a new material to the medical field: these fibers have been used in various medical applications for the past two decades. Abutaleb et al. (2021) have described various biodegradable nanofiber materials from natural resources for medical applications [145]. Nanotechnology is highly reliable in developing effective, scalable, and cheaper air filters for masks and respirators. Electrospun techniques have the capacity to produce a smaller pore size of several micrometers when compared to commercial filters. ES fibers capture smaller airborne particles [145,146]. In this study, a coronavirus aerosol test against an ES mask was carried out. A polypropylene fabric was electrospun with polyvinylidene fluoride PVDF_20_ and PVDF_30_. This layer was soaked into a polyelectrolyte poly(ethylenimine) (PEI) and poly(vinylphosphonic acid) (PVPA), both of which were positively or negatively charged to enhance the electrostatic attraction for virus removal. The diameter of this PVDF nanofiber was found to be 0.2–1.3 µm, but other commercial face masks and neck gaiters in this study showed fiber diameters of 5.7 ± 2.8 and 12.0 ± 1.0 µm. Usually, fibers with a larger diameter and pore size are less effective as an airborne particle filter, but those with a smaller diameter and pore size are highly effective in the filtration of aerosols. This was achieved by increasing the ES spinning time, which exhibited the highest removal of coronavirus aerosol up to 99.9% for PVDF_30_ and 99.1% for PVDF_20_ Figure 9 [147]. The reuse of the face mask is an important question in this pandemic. A study was carried out to identify the melt-blown filter used in the N95 face mask and the reusability of the nanofiber filter face mask with 75% ethanol treatment. It showed that there was a loss of filtering efficiency in N95 masks after ethanol treatment, but nanofiber filter masks showed promising results of the filtering capacity up to 97–99%, irrespective of the cleaning method [148]. Economically cheaper materials such as ES fabrics are highly recommended for mask production during this pandemic. These ES air filters are more competitive against other commercial filters on the market, and a portable ES apparatus can be helpful in the rapid preparation of ES masks at home and for minor populations [145]. Recently, various methods have been used to produce ES-based masks to combat the pandemic, and some findings are listed below in Table 3.

### 3.9. Robotics against COVID-19

Robotics is the most advanced research area developed to deal with conditions where humans cannot work or concentrate. Already, several studies have brought new developments to robotics, and now the pandemic has brought new ideas for using robots to manage various functions such as nursing [155], diagnostics and treatment [156], logistics management [157], etc. A cost-effective robot was designed based on the S4 concept of “sensing, smart, sustainable, and social features”. AI was adopted as one of the finest choices for improving robot intelligence. An AI algorithm was developed to assist in mask detection, crowd detection, and obstacle detection, along with a fuzzy logic controller that was used to prevent crashes against objects in its path, or instant multiple obstacles that could now be detected by humans. The most attractive objective in this Robocov robot is the sanitization module, which can be used in hospital cleaning using a UV lamp installed with this robot. This is far more useful for cleaning the COVID-19 patient ward in hospitals, as it avoids infecting human workers with the coronavirus. Robocov is further used for disinfection, identification of infected people by temperature sensors, symptom monitoring, and package delivery [158]. Future goals include (i) high-power batteries for Robocov for long-distance travel within cities; (ii) road signal detection systems; (iii) long-distance Google navigation system; (iv) automatic driving system; and (v) human face detection for package delivery.

## 4. Impact of COVID-19 Pandemic in Public Life

The pandemic has caused a great deal of effects on many aspects of human life, the economy, industry, etc. In numerous recent research studies, AI approaches have been used to develop ways of managing the effects of the pandemic.

### 4.1. AI in Utility Services

The recent research used a machine learning model, namely the Pandemic Electricity Consumption Scenario (PECS) model, for the prediction of energy usage in India. This model was used to effectively analyze and measure the impact of the pandemic on electricity consumption based on weather, econometrics, and social distancing in seven major states in India. The machine learning model predicted a 15 to 33% drop in consumption from March to May in 2020 during the complete lockdown period, and a 6 to 13% drop from June to August 2020 during the unlocked period, returning to normal in September 2020. The analysis also showed a drop in CO2 emissions of between 7 and 5% compared to previous years. This prediction can be used in future energy policy decisions [159]. A similar study was conducted in China using a comparative regressive and neural network model to identify the impact of the pandemic on electricity and petroleum demand. The model showed a downtrend in the demand for fuel consumption [160].

### 4.2. AI for Researchers

The search engines for the extraction of COVID-19-oriented publications from servers are more important for researchers. A powerful language model, BERT, is used by Google for extracting data [161]. A CORD-19 search and COVID-19 research explorer information retrieval (IR) system were analyzed for the study. CORD-19 was subjected to a language processing service, in Amazon Comprehend Medical (ACM), which is a natural language processing (NLP) service for extracting clinical data from unstructured text [161].

### 4.3. ML in Oil and Gas

The machine learning model of the pandemic oil demand analysis (PODA) predicted gasoline consumption in the USA. Since the oil price went negative due to the pandemic, the predictions were made based on public travel, their trip activities, and fuel usage. Later on, in October 2020, the prices gradually recovered, so this model predicts the demand for oil and its impact for a shorter duration [162].

### 4.4. AI-Based Decision-Making in the Hospital

Recent digital technologies were implemented in cardiology-based devices to collect and observe data. Machine learning (ML) was used to study large datasets from hospitals for clinical practice, and this was further used to identify complex heart problems and treatment strategies for COVID-19 patients [38]. There are various ML algorithms used to screen the structured data in hospitals, such as ensemble, support vector machine (SVM), hierarchical clustering, and topological data analysis, and for unstructured data, convolutional neural network (CNN), deep neural network (DNN), AdaBoost, and long short-time memory (LSTM). A DL model was proposed to identify which patient may receive more of a benefit from surgery in treating epilepsy [39]. AI assists in providing a strategic execution picture in operating theater surgeries by lowering risk and ensuring surgical success [163]. Cardiothoracic surgery, a fine example of AI-supported cognitive augmentation, requires a combination of a doctor team, highly advanced equipment, and special care [163].

### 4.5. Telemonitoring during COVID-19

The value of telemonitoring should be taken into account in the care and monitoring of patients in hospitals, and even in outlying areas nowadays. Various portable devices, such as a blood pressure cuff, glucometer, pulse oximeter, ECG + stethoscope, activity trackers, wearables, thermometer, etc., are now used by patients even at home. Aysha Shabbir et al. (2022) proposed an improved model for remotely monitoring a patient’s condition and to make decisions based on that condition using ML and DL models. In this process, the remote monitoring station’s patient monitoring device sends signals about higher and lower thresholds via the Internet using Cloud computing and IoT servers, which are relayed to a healthcare specialist. Later, primary/secondary care requirements are decided by specialists based on severity. This was framed by a pattern of sensing, transmission of data, interaction with the patient, and situational response [164]. In Italy, 23.2% of the population is 65 years of age or older, making it difficult to treat them during the COVID-19 pandemic. Therefore, they proposed a connected-care solution in the context of digital health, where the citizen or patient was kept in a high-priority center and supported with various integrated organizational measures. Further, in this recent organizational model, patient clinical information was shared with different healthcare workers involved in the treatment process. For this purpose, various healthcare models were combined to support patients with chronic illnesses. A remote/home healthcare Resilia app, a simple mobile phone application, was introduced by Italy healthcare, and guides users to identify nursing care, doctors, and other healthcare professionals for a quick response [165]. The northwest Tuscany region of Italy consists of local health units that have introduced territorial telemonitoring of chronic patients (Tel.Te.C.): telemedicine mobile applications since 2017. Both the patient and the healthcare professional receive a home monitoring kit and a professional monitoring kit, from which vital parameters such as temperature, heart rate, oxygen saturation, blood pressure, and weight can be measured; any parameter that is out of range raises an alarm for immediate action. Under compulsion, this platform has been used by 40 general physicians and 180 patients since March 2020. Patients with different stages of illnesses have been enrolled in this application, which has resulted in positive feedback from patients and doctors with reduced hospitalization and no mortality [166].

### 4.6. AI-Based Law in Various Countries

Various countries understand the importance of AI-based technology and their applications in the health sector and are moving forward to bring AI under national law. A proper legal channel is suggested by different counties to justify its safety and reliability in the healthcare and clinical sectors. Similarly, the FDA approved 350 AI- and ML-based equipment for use in the healthcare in the United States of America until 2021 [167]. Similarly, various countries implemented AI/ML laws in the area of data protection, AI robotics, decision support software, diagnosis and predicting patient conditions, COVID-19 tracking apps, etc. (see Table 4).

## 5. Discussion

In this paper, we highlighted the applications of AI and its recent developments in various fields in tackling the pandemic. We detailed the impact of AI in confronting coronavirus handling; secondary infections such as CAM, blood coagulation, blood stream infections, UTI, and antibiotic resistance; diagnosis and treatments in healthcare; along with prevention measures such as face mask detection, production, design, and robotics in public life. Even though its applications seem to be highly useful, it is still in the beginning stages and needs more room to improve and be implemented in different applications of healthcare to tackle the situation. Still, many hospitals in various countries are in a dilemma when choosing AI-driven solutions for screening and treatment. As a result, various countries enact laws and act to implement AI and ML in various fields. Because AI is currently used in a variety of fields, we highlighted some research gaps and challenges that AI is currently facing, which can be useful in future exploration.

The secondary infections are predominant in COVID-19 patients: CAM has been reported in various countries such as India, Iran, Italy, the USA, Brazil, and Egypt [14]. Novel therapies are under research for the treatment of CAM. Currently, there is no direct medicine for CAM control, but combination therapy with AmB and atorvastatin is more promising when treated against *R. arrhizus*. However, it should be studied in detail to deliver this combination to diabetes patients. The use of statins to treat CAM is being considered, but research is still in its early stage [189]. Coagulation problem is more frequent in COVID-19 patients after CAM. Pulmonary embolism and venous thromboembolism are more prevalent in hospitalized patients. Preventive measures such as observing platelet count, d-dimer marker, and C-reactive protein during admission will help in the earlier identification of coagulations. BSI infections with bacteria are more common in patients admitted to the ICU; around 14% of patients admitted to the ICU acquire BSI. The majority of superinfections are caused by carbapenem-resistant *K. pneumonia* bacteria in the respiratory tract, particularly in the ICU [88]. Similarly, UTI infections are more prevalent along with the BSI. UTI or CAUTI emerge in hospitalized COVID-19 patients, with the most prevalent infections arising from ICU-acquired bacterial infections. *Enterococcus* sp. and *E. coli* are predominant in UTI patients. It is an urgent situation to implement new methods to prevent CAUTI-based infections, and pre-planned antibiotics treatment will become more beneficial in saving lives [22]. Most of the above-mentioned circumstances occur due to the uptake of antibiotics without proper guidance by physicians. It is complicated to treat antibiotic-resistant bacteria, which could be more harmful to patients. Among various bacteria, *Pseudomonas* and *Stenotrophomonas* families are more virulent in ICU patients. In many cases, ICU-acquired AR bacteria are more critical to handle since they may be resistant to various families of antibiotics. Since microbial resistance to antibiotics is increasing every year, national recommendations of antimicrobial stewardship principles during the COVID-19 pandemic need to be followed to minimize hospital-acquired AR. To overcome the effects of a pandemic and reduce the burden on healthcare, the help of AI is inevitable.

ML, which uses huge healthcare datasets to train the program, is more accurate in predicting coronavirus outbreaks. The use of a neural network trained on an organized dataset to support machines involved in the diagnosis of diseases in healthcare is a vital approach. DL models can be used to analyze CAM, blood coagulation, BSI, UTI, and AR using images and clinical data from models such as XGBoost, HLNNC, CNN, and SVM. Furthermore, chest X-ray and CT scans using deep neural networks can study patient status and health conditions. Many advanced technologies are used to confront the pandemic, but AI [190], blockchain [191], open-source technologies, telehealth technologies [192], 3D printing [193], gene editing technology [194], nanotechnology [145], synthetic biology [195,196], and robots [158] are the most significant. These advanced technologies are combined with various models to produce promising outcomes, and further enhancements are needed to train with large public and hospital datasets to combat the pandemic.

The telemonitoring of patients is especially beneficial during the COVID-19 pandemic, because, according to numerous publications, patients have the greatest potential for viral transmission during the incubation period. Due to their increased susceptibility to infection, older persons frequently require general or urgent hospital care. In some circumstances, even people who have recovered from an infection can spread it to others. As a new era of homecare systems, telehealth monitoring is a breakthrough in patient health condition monitoring from their homes, minimizing direct contact between an infected person and a healthcare practitioner. Introducing digital technology for health monitoring in the patient’s home improves the homecare system, which could monitor even simple movements, wake-up pattern, medical alerts, and nursing staff on demand [197,198]. In order to combat the pandemic, telemonitoring tools such as the Resilia app, Tel.Te.COVID-19 platform, and the Italian linked-care solution are highly valued, considering that these platforms may improve the lives of elderly people and patients who are unable to take care of themselves. Every new innovative technology has its own drawbacks, which should be considered in order to avoid any complications for users. Considerations should be made for elements such as software repeatability, data privacy and security, and civil responsibility brought on by software or programs for health monitoring. Who is liable if any AI or monitoring software fails to provide a positive outcome in a treatment or poses a health risk to the patient? This is because many people, including the producer, developer, distributor, programmer, doctor who typically recommends it, and patient who used it, were involved in its creation. Similar problems are frequently seen in other AI application sectors. The legal systems of various nations should presumably specify clear decision-making pathways that are guaranteed for the patient when employing this type of AI-based telemonitoring software.

Though we have discussed the recent nanofiber membrane technologies in fighting COVID-19, our primary shield against the virus remains the face mask. By considering further modifications to the nanofiber mask, such as surface charge, hydrophobicity, and antiviral-embedded nanoparticles, we can manage the spread of the virus to the maximum. Mask reusability, a major question, now has a positive answer: “yes”, but only with nanofiber masks. The biopolymer is the best choice of nanofiber mask material since it can be reused and is eco-friendly; we can avoid single-use disposable masks for the sake of the environment and a lack of demand.

➢ A face recognition and attendance project is in the developmental stage, and a face mask recognition system must be included to detect a violation committed by a specific person in a closed office environment and send an alert to management.
➢ The Center for Disease Control and Prevention (CDC) maintains a large database of “COVID-19 Science Update Database” published and pre-print databases which are easily accessible for any AI-based healthcare articles. Similarly, Lawrence Berkeley developed a COVID-19 literature search powered by advanced NLP algorithms, which uses AI to search attributes such as keyword, topic, title, year, and so on.
➢ Another central data service for documents is CORD-19 and LitCovid, with a regular update on COVID-19 research outputs. These databases encourage the scientific community to access free articles, which helps in developing novel research ideas and outputs.
➢ The promising results obtained from various drug makers’ companies’ AI-based technologies give hope to end the pandemic, but the real question is why these drug companies are having such difficulty sharing their data with low-income countries to end the pandemic. Until this happens, COVID-19 may repeatedly invade developed nations with various mutated stains.
➢ Hospitals need to adopt recent AI technologies for analyzing radiographic images of patients with COVID-19, to reduce the burden on doctors. Telemonitoring and speech and text recognition using NLP will improve doctor–patient interactions in remote treatment methods.
➢ AI offers doctors and health workers the ability to store and handle or share patient records very safely and securely through Cloud platforms such as Google Cloud AI, Amazon AI services, Microsoft Azure AI, IBM Watson Studio, ref. [199] H2O.ai, TensorFlow, DataRobot, Wipro Holmes AI and automation platform, Salesforce Einstein, Infosys Nia, and others that can be accessed through AI-infused technology.

At the time of writing this manuscript, the WHO has approved 11 vaccines for use in emergencies around the world [200]. We have been forced to identify various effective control measures and search for effective medicines and vaccines due to the increase in the number of cases and the impact on the current global economy. In this context, earlier detection of COVID-19, prediction, and hospital management are vital for controlling the cases and mortalities. AI has become a boon for the current situation in addressing the pandemic and providing a ray of hope in various healthcare and technological areas.

## 6. Conclusions

The collection of findings made available here demonstrates that AI-based ML and DL models can be extremely helpful in diagnosing and forecasting a variety of healthcare issues connected to COVID-19 infections, as well as assisting healthcare professionals in monitoring and making decisions. To locate and gauge the severity of an illness, numerous separate algorithms are employed. However, when compared to AI-based tools, it is widely acknowledged that human intellect is a pioneer in decision-making. The observations and diagnoses made by doctors and medical personnel in emergency situations are incomparable to those made by computers; however, AI can work in tandem with health professionals to lighten their workload during this urgent pandemic period. Few options are given to the medical professionals at each level of their diagnosis and treatment.

## Figures and Tables

**Table 1:** Summary of studies using machine language for prediction of antibiotic resistance for clinical decision-making.

Task	Input Data	Primary Models Used in the Study	Comparisons Models Used/Tested	Peformance/Accuracy	Comments/Limitations	References
Antibiotic resistance prediction in patients	11,496 antimicrobial susceptability datasets from laboratory information system, internal medical ward, public hospital, Greece	stack ensemble (Microsoft Azure AutoML)	VotingEnsemble, MaxAbsScaler, LightGBM, Sparse Normalizer, XGBoost Classifier	AUCW is 0.822 and 0.850 Accuracy rate is 0.770	Study uses 499 patients’ data. Data scientist is needed at this stage for pre-processing, feature selection, and final analysis. More data training needed to obtain more accurate results.	[107]
Prediction of antimicrobial resistance in *Acinetobacter baumannii*, *Mycobacterium tuberculosis*, and *Streptococcus pneumoniae*	K-mer representation of bacterial genomic data	random forest	Adaboost, logistic regression, deep learning	80–92%	Lower data give less accuracy and higher number of data give elevated accuracy. Not possible to use by laboratories and hospitals having small datasets.	[109]
Predicting antibiotic resistance in hospitalized patients	5590 instance datasets with different variables from general hospital laboratory, Greece	WEKA framework ML, run under Java platform	J48 algorithm, random forest, multinomial logistic regression, kNN algorithm, multilayer perceptron (MLP)	ROC area of 0.758 and accuracy of 75.8%	Low accuracy, limited dataset, and less clinical attributes. Increased dataset can give accurate results.	[110]
Prediction of antibiotic resistance in hospitalized patients	16,000 antibiotic resistance tests of electronic medical record (EMR)	Ensemble of 3 models: Lasso logistic regression, neural networks, gradient boosted trees	Independent algorithms:Lasso logistic regression, neural networks, gradient boosted trees	Combined algorithm: 0.821, Xgb: 0.82, Lasso: 0.82, dnn: 0.803, auROC score was 0.8–0.88	Bacterial details can increase AUROC score if included, additional information needed to improve accuracy such as antibiotics used prior to admission, microbiome composition, diet, and exercise.	[111]
Prediction of antimicrobial resistance from ICU patients in *Pseudomonas* and *Enterococcus*, *Stenotrophomonas*	Dataset of 32,997 collected from health information system of 2630 patients, University Hospital of Fuenlabrada, Spain	Logistic regression, *K*-nn, decision trees, random forest, multilayer perceptron		AMG: 82.2 ± 1.7, CAR: 79.6 ± 2.1, CF4: 74.9 ± 2.1, PAP: 77.1 ± 1.7, POL: 68.5 ± 7.0, QUI: 88.1 ± 1.6	Accuracy differs based on various antibiotics and bacterial species; upgradation yet to undergo based on mechnical ventilation, bed; sepsis patients in ICU are to be considered.	[106]
Prediction of Carbapenem-resistant *Klebsiella pneumoniae*	46 Carbapenem-resistant *Klebsiella pneumonia* (CRKP) isolated from hospital patients	random forest	Logistic regression, Naïve Bayes, nearest neighbors, support vector machine	Accuracy: 97%, Carbapenem-resistant identification: 93%	Small sample size and limited data of CRKP.	[112]
Prediction of antimicrobial phenotype resistance of *Staphylococcus aureus*	K-mer representation of bacterial genomic data	Random forest, SVM, XGBoost		Among 10 anitibiotics used in the study, AUC was recorded between 82.02% for Linezolid and 96.13 for vancomycin. Cefoxitin registers AUC of 92.65% with sensitivity of 94% and major error 6.82%	Study uses 466 whole genome sequencing results to predict the antimicrobial resistance.	[108]

**Table 2:** Artificial Intelligence-based models for detecting face masks.

AI Models	Description of the Model	Accuracy	Image Dataset Sources	Reference
Hybrid deep transfer model	This model consists of SVM, decision tree, and accurate methods to detect face masks.	Achieved 99.64% on test	RMFD: real-world masked face dataset, SMFD: stimulated masked face dataset, random face in crowd	[135]
Inceptionv3 CNN	This module consists of 22 layers deep from GoogleNet to increase accuracy; this CNN detects persons without a mask.	Achieved 99.9% accuracy	Simulated masked face dataset was used in this study	[136]
Facemasknet model	This model uses DL model to identify masked face, properly masked face, and no mask face.	This model achieved 98.6% accuracy	Datasets used in this study are MFDD: masked face detection dataset, RWFCD: real-world face recognition dataset, SMFRD: simulated masked face recognition dataset	[137]
EfficientNet model	CNN-driven EfficientNet architecture is applied in this method and can be used for real-time detection of mask.	Achieved accuracy of 97.12%	Openly available face mask detection dataset	[138]
Deep learning model vgg16	Trained with 2 datasets, it works well with medium and small datasets.	Accuracy of 96.50% was achieved	Two datasets with 1484 and 7200 images are used	[139]
DTLMV2 (deep transfer learning MobileNetV2)	This model uses a lightweight CNN which requires less computing power and is easily attached to computer vision and mobile system.	This model gains accuracy of 97.01% at validation data and 98% accuracy on training data	Crowd dataset with 7514 images is used	[140]
Deep Masknet framework	This model uses both face mask detection and masked facial recognition.	Obtained 100% accuracy on face mask detection and 93.33% of masked facial recognition	Mask detection and masked facial recognition (MDMFR) dataset	[141]

**Table 3:** Overview of various mask materials produced through nanofiber/material technologies to confront the pandemic.

Type of Mask	Material Used	Filtering Size/Efficiency%	Characteristics	Reference
ML algorithm-based respirator	Elastic fiber membrane (EFM)	Up to 2.5 µm	Controlled mechanical stretching and relaxation of filter, response to slow and fast physical activity, cheaper material.	[144]
N95	Electrostatic non-woven polypropylene	Up to 0.3 µm	High filtering efficiency of virus/bacteria, prevents air and droplets penetrating the edges.	[149]
Mask with nanofiber filter	Polyvinylidene fluorid (PVDF_30_) and (PVDF_20_)	≤2.7 μm	Captures up to 99.9% of coronavirus aerosol.	[147]
Nanoparticles-coated non-woven surgical mask	Copper nanoparticles (CuNPs)	99.37%	Photocatalytic and photothermal properties, reusable, self-cleaning ability, disposable.	[150]
Surgical mask with graphine	Melt-blown non-woven fabrics (MNF) and graphene layer with electric and thermal conductivity	99.8%	Removal of virus/microbes by electrothermal method, high removal efficiency up to 10 cycles.	[151]
Nanofiber mask	Piezoelectric electrospun poly (l-lactic acid) (PLLA) nanofibers	>99%	Respiration electrifies the mask, long stable filter is humidity resistant, autoclavable, and degradable around 50 days.	[152]
N95 respirator nanofiber mask with copper nanoparticles	Nylon polymer fiber with copper nanoparticles	-	Antiviral and antimicrobial properties.	[153]
3D-printed nanofiber-based mask	Combined silver and copper nanoparticles	99.5%	Antimicrobial, high polarity, heat-insulating properties.	[153]
Nanoporous hard mask	Flexible silica fabricated with reactive ion etching process on polymeric membrane	Up to 5 nm	Reusable, high filtration efficiency, hydrophobic, antifouling, self-cleaning.	[154]

**Table 4:** Laws and regulations implemented based on AI healthcare in various countries past and present regarding the pandemic.

Country	Law/Regulation	Purpose of This Law	Date Effective
USA	No Vaccine Passports Act [168]	Relaxing the restrictions of forcing vaccine certificate	4 August 2021
The Netherlands (Red Cross)	510 Data Responsibility Policy [169]	Data protection	12 November 2018
United Kingdom	Contact-tracing app (General Data Protection Regulation (UK GDPR) and Data Protection Act (DPA) 2018) [170]	Data protection and digital COVID tracking app	May 2020
European Union	General Data Protection Regulation [171]	Data protection of public and health records	27 April 2016
European Union	Medical Devices Regulations 2017/745 (MDR) [172]	Protection of patients from medical device, protection of produced data using this device	5 April 2017
European Union	The 2017/746 In Vitro Diagnostic Medical Devices Regulation (IVDR) [173]	Protection of patient health and users, quality and safety of in vitro medical devices	25 January 2022
European Union	Regulation of the European Parliament and of the council laying down harmonized rules on AI (Artificial Intelligence ACT) and amending certain union legislative acts [174]	Facilitate and creating innovation in AI, creating trusted AI applications	21 April 2021
European Union	Civil Law Rules on Robotics [175]	Implementation of AI robotics	16 February 2017
Singapore	Personal Data Protection Act 2012 [176]	Data protection	31 December 2021
Australia	Therapeutic Goods (Medical Devices) Regulations 2002 [177]	Clinical decision support software	25 February 2021
China	Notice of the State Council Issuing the New Generation of Artificial Intelligence Development Plan. State Council Document. No. 35. 2017 [178]	Healthcare and management	8 July 2017
Kingdom of Saudi Arabia	Guidance on Software as a Medical Device/SFDA MDS-G23 [179]	AI- and BigData-based medical software to diagnose and predict patient conditions	27 April 2021
Russia	Development of AI in healthcare up to 2030, approved on 10 October 2019, No. 490 [180]	Software as medical device in healthcare	10 October 2019
South Korea	Medical Devices Act No. 15945, 11 December 2018 [181]	Software as medical device in healthcare	11 December 2008
Singapore	Standalone Medical Mobile Applications (SaMD) and Qualification of Clinical Decision Support Software (CDSS) [182]	Clinical decision support software	19 July 2021
China	Cybersecurity Law of the People’s Republic of China [183]	To preserve cyberspace sovereignty and national security	7 November 2016
Malaysia	Medical Device Act 737-2012 [184]	Medical device, software regulation in healthcare	30 January 2012
Emirate of Abu Dhabi	Artificial Intelligence (AI) in the Healthcare Sector of the Emirate of Abu Dhabi, Policy/AI/0.9, Version 0.9 [185]	Health system monitors, analysis, and public health observation	30 April 2018
Canada	Digital Charter Implementation Act, 2022 (Bill C-27) [186]	Protection of personal information, data, and health records, along with any serious direct cause to patients by AI	16 June 2022
Brazil	LGPD–General Personal Data Protection Law (Federal Law no. 13,709/2018) [187]	AI regulation in health sector of Brazil	14 August 2018
Brazil	Brazilian Artificial Intelligence Bill (Bill No. 21/2020) [188]	Development and applying of AI in various sectors of Brazil	29 September 2021

## Data Availability

No new data were created or analyzed in this study. Data sharing does not apply to this article.
